# Population Based Study of 12 Autoimmune Diseases in Sardinia, Italy: Prevalence and Comorbidity

**DOI:** 10.1371/journal.pone.0032487

**Published:** 2012-03-02

**Authors:** Claudia Sardu, Eleonora Cocco, Alessandra Mereu, Roberta Massa, Alessandro Cuccu, Maria Giovanna Marrosu, Paolo Contu

**Affiliations:** 1 Dipartimento di Sanità Pubblica, Università di Cagliari, Cagliari, Italy; 2 Centro Sclerosi Multipla, Dipartimento di Scienze Cardiovascolari e Neurologiche, Università di Cagliari, Cagliari, Italy; Wayne State University, United States of America

## Abstract

**Background:**

The limited availability of prevalence data based on a representative sample of the general population, and the limited number of diseases considered in studies about co-morbidity are the critical factors in study of autoimmune diseases. This paper describes the prevalence of 12 autoimmune diseases in a representative sample of the general population in the South of Sardinia, Italy, and tests the hypothesis of an overall association among these diseases.

**Methods:**

Data were obtained from 21 GPs. The sample included 25,885 people. Prevalence data were expressed with 95% Poisson C.I. The hypothesis of an overall association between autoimmune diseases was tested by evaluating the co-occurrence within individuals.

**Results:**

Prevalence per 100,000 are: 552 rheumatoid arthritis, 124 ulcerative colitis, 15 Crohn's disease, 464 type 1 diabetes, 81 systemic lupus erythematosus, 124 celiac disease, 35 myasthenia gravis, 939 psoriasis/psoriatic arthritis, 35 systemic sclerosis, 224 multiple sclerosis, 31 Sjogren's syndrome, and 2,619 autoimmune thyroiditis . An overall association between autoimmune disorders was highlighted.

**Conclusions:**

The comparisons with prevalence reported in current literature do not show outlier values, except possibly for a few diseases like celiac disease and myasthenia gravis. People already affected by a first autoimmune disease have a higher probability of being affected by a second autoimmune disorder. In the present study, the sample size, together with the low overall prevalence of autoimmune diseases in the population, did not allow us to examine which diseases are most frequently associated with other autoimmune diseases. However, this paper makes available an adequate control population for future clinical studies aimed at exploring the co-morbidity of specific pairs of autoimmune diseases.

## Introduction

The continued surveillance of autoimmune diseases contributes greatly to the study of their aetiology and temporal trends, and to the understanding of the relationship among these disorders. Such [1]. Several studies on the epidemiology of autoimmune diseases performed within the past several decades are discussed in a recent review that highlights the importance of future studies that could address limitations identified in the current literature [1].

The critical factor in studying the prevalence of autoimmune diseases is the availability of a systematic and unbiased source of data that are representative of the general population [1]. Routine registration systems, like death statistics and hospital admission and discharges, provide only a partial picture of the prevalence of autoimmune diseases, because these diseases are characterized by a low fatality and hospitality [1]. Collection of data through population-based studies is the most suitable method for obtaining prevalence data without ascertainment problems, though these studies require considerable resources, in fact they are often based on self reported data. However, in the case of autoimmune diseases, which include rare diseases and diseases with considerable clinical heterogeneity and complex case definitions, the collection of data through self reporting involves a high probability of referral bias [1]–[3]. The few studies performed on the general population are based on laboratory screening and consequently focus only on autoimmune diseases detectable through laboratory tests [11], [17], [18]. Additionally, their results include asymptomatic forms of disease.

The limited availability of prevalence data based on a representative sample of the general population is a possible reason for the lack of definite evidence on the co-morbidity of autoimmune disorders [1], [2], [3], [4]. Several studies support the hypothesis that there is a common aetiology for autoimmune diseases. These studies usually assess the prevalence of a second autoimmune disease in samples of patients already suffering from a first one and compare these prevalence data with those of the patients' spouses or first degree relatives. A higher susceptibility to a second autoimmune disease is regarded as an indication of potential common pathogenic mechanisms among autoimmune diseases. The main limitation of these studies is the lack of an adequate control population [3]. The prevalence obtained in spousal or familial controls is based on self reported data that may be affected by several biases (e.g. gender, age, educational level, and confusion between diseases with similar names). In addition, the design of these studies allows the evaluation of the co-morbidity of three or four diseases at most. This limit is due to the limited number of patients who already have an autoimmune disorder and the low frequency of concomitant autoimmune diseases. The limited number of diseases considered in studies about co-morbidity makes it difficult to evaluate the hypothesis that there is an overall association between autoimmune diseases [1], [4].

This paper aims to contribute to the study of autoimmune diseases through three specific objectives. First, we describe the prevalence of the main autoimmune diseases in a representative sample of the general population in the South of Sardinia, Italy. Next, we assess gender differences in prevalence of autoimmune diseases. Last, we test the hypothesis of an overall association among the main autoimmune diseases.

## Methods

### Study design

The design of this study is based on the Italian National Health System (INHS), which makes the General practitioners (GPs) a potential source of data for epidemiological studies.

The INHS covers the entire resident population, irrespective of social status or pre-existing health conditions, and provides each citizen with the service of a GP free of charge. Each citizen is required by law to choose one GP from a list of at the Local Health Agency. The choice of GP is mainly influenced by the proximity of the GP's office to the citizen's place of residence rather than by the citizen's health status. GPs provide primary care, prescribe pharmaceuticals and diagnostic procedures, and refer patients to specialists and hospitals. Drugs for chronic diseases are given free of charge by the INHS, provided that they are prescribed by the GP. According to Italian law, a GP can prescribe a drug “ free of charge” for a chronic disease only if the diagnosis is performed and certified by INHS specialist. As a result of this system, GPs are the main source of healthcare in Italy.

This study was carried out by the Department of Public Health of the University of Cagliari-Italy, in collaboration with 21 GPs who are members of the “Italian Society of General Medicine” (SIMG).

Each GP involved in the research had recorded patient data for at least one decade using the software “Millewin”. There is no reasonable reason to suppose that people followed by these GPs have health status different from that of the people followed by GPs who do not register data with millewin. The diagnoses of autoimmune diseases were performed by INHS specialists with residency in different specific medical fields, and then entered into the database by the GPs. entered into the database only after the confirmation of the diagnosis by an INHS specialist. The validity of diagnosis is guaranteed by specialists' daily clinical practice in public health system, adherence to National Programmes on Clinical Guidelines and Continuing Medical Education programmes.

The Millewin software aims to make easily available to the GP the main clinical data crucial for the care management, and offers the following features as recording data (e.g. demographics characteristics, ICD-IX diagnosis codes, ongoing therapy, prescribed medications, adverse reactions, diagnostics, requests of various kinds), management of drugs prescription, and an update version of the Regional Therapeutic Formulary. This study focused on a selected group of 12 diseases with chronic course and a certain autoimmune component and that, also for unanimous consensus among researchers and GPs, could be characterized by well defined shared diagnostic criteria.

The syntax for extracting data from software Millewin, set up by researchers and GPs, included demographic data and data on the following diseases: rheumatoid arthritis (ICD-9: 714.0/00), ulcerative colitis (ICD-9: 556.9/00), Crohn's disease (ICD-9: 555.9/00), type 1 diabetes (ICD-9: 250.01/00), systemic lupus erythematosus (ICD-9: 710.0/00), celiac disease (ICD-9: 579.0/00), myasthenia gravis (ICD-9: 358.0/00), psoriasis/psoriatic arthritis (ICD-9: 696.1/00, 696.0/00), systemic sclerosis (ICD-9: 710.1/00), multiple sclerosis (ICD-9: 340.0/00), Sjogren's syndrome (ICD-9: 710.2/00), and autoimmune thyroiditis (ICD-9: 245.2/00).

### Sample

The survey was performed in 2009 in Sardinia, Italy, which is a Mediterranean island with a population of 1,671,000 inhabitants.

Data collection covered a representative sample of the southern part of the regional territory, provinces of Cagliari and Carbonia-Iglesias with 700,000 inhabitants, and included the metropolitan area of Cagliari, the 2 urban towns of Carbonia and Iglesias, and the 3 semi-urban villages of Narcao, Masainas, and Sant'Anna Arresi. The metropolitan area of Cagliari accounts for half of the population, the urban towns accounts for 30% of the population, and the small villages for the 20%. The considered sample covers proportionally the three different settlements of the southern part of the region, thus preventing the possibility of a systematic error regarding to the characteristics of the people included in the sample. In fact 10 out of 21 GPs are from the metropolitan area, 7 are from the urban towns, and 4 from the semi-urban villages. The research focused on those individuals who were between 15 and 89 years old. Children under 15 years of age were excluded because they are normally treated by paediatricians. The data were extracted, in July 2009, by each GP from his or her personal database, cleared of identifying information (that were substitute with a code created taking into account the first letter of name and surname, the place and date of birth, the sex, the place of residence), and provided to the researchers.

The sample included 25,885 people, 14, 167 of whom were women and 11,718 of whom were men.

### Statistical analysis

Prevalence data were expressed as cases per 100,000 population, and 95% confidence intervals were calculated according to the Poisson distribution. The total number of people between 15 and 89 years of age registered with GPs involved in this study was used as denominator for prevalence.

Gender differences in the prevalence of autoimmune diseases are examined using Fisher Chi square test.

The hypothesis that there is an overall association between the autoimmune diseases was tested by evaluating the co-occurrence of autoimmune diseases within individuals. The observed and expected numbers of people with zero, one, or more than one co-occurring autoimmune diseases were compared using Chi square test.

The number of expected cases was calculated, on the basis of the prevalence values of each disease according to the formulae described below.

The probability of not having any autoimmune disease, (P_0_), has been defined as the product of the probabilities of not having each examined disease. It is calculated as:

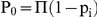
where p_i_ is the prevalence of each examined disease.

The probability of having only one autoimmune disease, (P_1_), has been defined as the sum of the products of the probability of having each specific disease and the probabilities of not having the other diseases. It is calculated as:

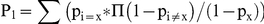
where p_i_ = x is the prevalence of a specific disease, whereas p_i≠x_ is the prevalence of the other diseases.

The probability of having more than one autoimmune disease, (P_>1_), is estimated as:

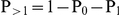



## Results

Table 1 shows the population distribution according to the presence or the absence of autoimmune diseases. Overall, the prevalence of autoimmune diseases was 5.0% with a 95% confidence interval of 4.7%–5.3%. More specifically, among the 1,300 people with autoimmune diseases, 95.6% were affected by one autoimmune disorder while the remaining 4.4% were affected by two autoimmune disorders. Among women the prevalence of people with autoimmune disorder was 6,9% . Among the 981 females with autoimmune diseases 95.2% were affected by one autoimmune disorder, whereas the remaining 4.8% were affected by two autoimmune disorders. Among men the prevalence of people with autoimmune disorder was slightly higher than it was among women (2,8%). Among men with autoimmune diseases, 96.9% were affected by one autoimmune disorder, whereas the remaining 3.1% were affected by two autoimmune disorders.

**Table 1:** Absolute frequencies of people with zero, one, or more than one autoimmune diseases.

	Number of autoimmune diseases		
	0	1	>1
**TOTAL SAMPLE (N = 25885)**	24,585	1,243	57
**WOMEN (N = 14167)**	13,186	934	47
**MEN (N = 11718)**	11,399	309	10

The prevalence of each autoimmune disease is shown in Table 2. The highest prevalence was observed for autoimmune thyroiditis. Myasthenia gravis, systemic sclerosis, Sjogren's syndrome and Crohn's disease were the least frequent diseases. The prevalence of autoimmune disease according to gender is reported in Table 3. Among women, the highest prevalence value was observed for autoimmune thyroiditis, followed by psoriasis/psoriatic arthritis, rheumatoid arthritis, type 1 diabetes and multiple sclerosis. Among men, the highest prevalence was observed for psoriasis/psoriatic arthritis, followed by type 1 diabetes, autoimmune thyroiditis and rheumatoid arthritis.

**Table 2:** Prevalence of each autoimmune disease per 100000 people.

	Prevalence per 10^5^	95% C.I.
**Autoimmune thyroiditis**	2,619	2426–2824
**Psoriasis/psoriatic arthritis**	939	824–1065
**Rheumatoid arthritis**	552	466–651
**Type 1 diabetes**	464	384–554
**Multiple sclerosis**	224	170–290
**Ulcerative colitis**	124	85–175
**Celiac disease**	124	85–175
**Systemic lupus erythematosus**	81	50–124
**Myasthenia gravis**	35	16–66
**Systemic sclerosis**	35	16–66
**Sjogren's syndrome**	31	13–61
**Crohn's disease**	15	4–40

**Table 3:** Prevalence of autoimmune diseases according to gender.

	WOMEN		MEN		p value
	Prevalence per 10^5^	95% C.I.	Prevalence per 10^5^	95% C.I.	
**Autoimmune thyroiditis**	4376	4039–4735	495	376–640	<0.001
**Psoriasis/psoriatic arthritis**	776	638–936	1135	950–1345	0.003
**rheumatoid arthritis**	741	606–897	324	229–445	<0.001
**Type 1 diabetes**	424	323–545	512	391–659	0.31
**Multiple sclerosis**	296	214–401	137	78–222	0.008
**Ulcerative colitis**	127	75–201	119	65–200	>0.99
**Celiac disease**	212	143–302	17	22678	<0.001
**Systemic lupus erythematosus**	148	92–227	0	0–31	<0.005
**Myasthenia gravis**	42	16–92	26	5–75	0.53
**Systemic sclerosis**	56	24–111	9	0–48	0.05
**Sjogren's syndrome**	42	16–92	17	22–62	0.31
**Crohn's disease**	14	2–51	17	2–62	>0.99

The prevalence values of autoimmune thyroiditis, rheumatoid arthritis, multiple sclerosis, celiac disease and systemic lupus erythematosus were significantly higher among women than among men. The prevalence of psoriasis/psoriatic arthritis was significantly higher among men than among women. The prevalence of the other autoimmune diseases was not significantly different between men and women.

Data about the co-morbidity of autoimmune diseases are shown in Table 4. The statistical analysis of the co-morbidity of autoimmune diseases highlights the fact that the number of people with more than one autoimmune diseases was significantly higher than the number expected under the null hypothesis that there is no association between autoimmune disorders. This finding was confirmed both in women and men. In order to verify whether the higher than expected number of people with more than one autoimmune diseases was only due to the high frequency of autoimmune thyroiditis in the sample under investigation, the analysis of co-morbidity was repeated excluding from the sample those people who were affected by this disorder. This analysis still indicated a significantly higher than expected number of people with more than one autoimmune disorder (p<0.001).

**Table 4:** Co-morbidity of autoimmune diseases: number of observed and expected people with zero, one and more than one autoimmune disease.

	Number ofAutoimmuneDiseases	NumberObserved	NumberExpected	p value
**TOTAL SAMPLE**	**0**	24,585	24,553	<0.001
**(N = 25885)**	**1**	1,243	1,308	
	**>1**	57	24	
**WOMEN**	**0**	13,186	13,161	<0.001
**(N = 14167)**	**1**	934	983	
	**>1**	47	22	
**MEN**	**0**	11,399	11,392	0.001
**(N = 11718)**	**1**	309	322	
	**>1**	10	3	

## Discussion

This paper contributes to the study of autoimmune diseases by defining the prevalence of the most common autoimmune diseases in a representative sample of the general population in South Sardinia, Italy, and investigating the co-morbidity between autoimmune disorders that affect different organs. To our knowledge, this is the first population-based study that investigates a consistent number of autoimmune diseases in a sample of the general population [1]. Autoimmune diseases are complex disorders caused by a combination of genetic susceptibility and environmental factors [3], [4], [5]. The island of Sardinia provides an ideal setting to investigate autoimmune diseases because of the genetic background of its inhabitants, who appear to be prone to these disorders [5], [6].

The unbiased source of data is the main strength of this research. Data were obtained from a database of 21 GPs who systematically recorded their patient data for at least one decade, and the appropriateness of each diagnosis was confirmed by a specialist. Furthermore, the reliability of the data presented in this paper can be confirmed by a comparison between the prevalence of type 1 diabetes and multiple sclerosis as determined on the basis of the data under investigation and the prevalences of these diseases obtained in previous population based studies performed in Sardinia [5], [7], [8]. According to the present research, the prevalence of type 1 diabetes is 464 cases per 100,000 people with a 95% C.I. ranging from 384 to 554. This estimate agrees with that obtained in a previous study, which found a prevalence of 459 cases per 100,000 people with a 95% C.I. ranging from 407 to 517 [5], [8]. Similarly, the present data on the prevalence of multiple sclerosis overlap with those estimated in a recent study, performed in the south-western part of the regional territory, which indicate a prevalence of 210 cases per 100,000 people with a 95% C.I. ranging from 168 to 234 [7].

This study showed that 5% of the general population is affected by one or more autoimmune diseases. These results agree with the data in the current literature that indicate an overall prevalence of autoimmune diseases in the range of 4%–5% [9], [10].

Overall, the prevalence was higher in women (7%) than in men (3%), confirming the existing evidence [10], [11].

According to our data, the autoimmune thyroiditis is the most widespread autoimmune disease with a prevalence value that is a reliable estimate of the symptomatic forms of disease, but it does not cover all of the asymptomatic cases, because GPs usually only investigate the presence of antithyroid-antibodies in people with clinical symptoms or with a family background of this disease. This prevalence is higher than what has been reported in hospital-based study, and it is similar only to prevalence values obtained in the United Kingdom [1], [9], [12].

Psoriasis/psoriatic arthritis and rheumatoid arthritis are the next most common autoimmune disorders, and their prevalence rates appear to be consistent with those reported in the current literature [1], [13], [14].

The prevalence values for type 1 diabetes, and multiple sclerosis confirm that Sardinia is among those regions with the highest prevalence of these disorders [5], [15].

The prevalence of ulcerative colitis, is lower than that observed in a Danish hospital-based study, whereas it appears to agree with the prevalence observed in Hungary [1], [9], [10], [16]. The rate of celiac disease is lower than the 7% prevalence observed in Italy through a screening program, but is similar to the values observed in the Danish hospital-based study and in a Greek study [9], [10], [17], [18], [19].

The prevalence of systemic lupus erythematosus is in line with prevalence data reported for Italy [16]. The prevalence of Myasthenia Gravis appears to be slightly higher than values observed in other population based surveys, but in contrast to the published literature, it is not different between men and women [9], [10], [20], [21], [22], [23]. The prevalence of systemic sclerosis similar to values reported in the current literature for European countries [9], [10], [24], [25]. Similarly prevalence of Crohn's disease, 15 cases per 100,000, is comparable to values observed in Bosnia and Hungary [16], [26]. The prevalence of Sjogren's syndrome, 31 cases per 100,000 people, is similar to that obtained in a Danish hospital-based study, but is lower than the value obtained in other population based studies [9], [10], [27].

These data on the prevalence of each autoimmune disease are valuable for the public health system, because they may allow an accurate evaluation of the burden of the diseases. The particular genetic background of the population under investigation suggests caution in generalizing these results to a world-wide level. In any case, these data provide a picture of the relative burden of the studied diseases.

With regard to the co-occurrence of autoimmune diseases within individuals, the present study highlights an overall association between autoimmune disorders, both in women and in men. According to our results, people already affected by a first autoimmune disease have a higher probability of being affected by a second autoimmune disorder. Our results agree with the hypothesis that there is a potential common pathogenic mechanism among autoimmune diseases [1], [28]. In the present study, the sample size, together with the low overall prevalence of autoimmune diseases in the population, did not allow us to examine which diseases are most frequently associated with other autoimmune diseases. However, this paper indirectly contributes to the achievement of this goal, because the data on the prevalence of each autoimmune disease make available an adequate control population for future clinical studies aimed at exploring the co-morbidity of specific pairs of autoimmune diseases.

Finally, this survey confirms the potential role of GPs in disease surveillance, offering an example of how a GPs' databases can be used for prevalence studies. To avoid the potential information bias of this source of data, the full involvement of GPs from the planning phase is the essential prerequisite because it guarantees the accuracy and completeness of the diagnosis. In Italy, as in several other countries, GPs are the main health care providers for citizens, and therefore they could be a valuable source of data that should be further explored [29], [30], [31].
